# Public and private pregnancy care in Reggio Emilia Province: an observational study on appropriateness of care and delivery outcomes

**DOI:** 10.1186/1471-2393-14-72

**Published:** 2014-02-17

**Authors:** Laura Bonvicini, Silvia Candela, Andrea Evangelista, Daniela Bertani, Morena Casoli, Annarella Lusvardi, Antonella Messori, Paolo Giorgi Rossi

**Affiliations:** 1 Servizio Interaziendale di Epidemiologia, Azienda Unità Sanitaria Locale and IRCCS, Arcispedale Santa Maria Nuova, Reggio Emilia, Italy; 2 Unit of Clinical Epidemiology, Azienda Ospedaliera Città della Salute e della Scienza di Torino, Turin, Italy; 3 UO Salute Donna e Infanzia, AUSL of Reggio Emilia, Reggio Emilia, Italy; 4 Dipartimento Cure Primarie, AUSL of Scandiano, Scandiano, Italy; 5 Programma Materno Infantile, AUSL of Reggio Emilia, Reggio Emilia, Italy; 6 Epidemiology Unit, Local Health Trust of Reggio Emilia, via Amendola 2, Reggio Emilia, Italy

**Keywords:** Pregnancy care, Midwife, Caesarean section, Ultrasound scan

## Abstract

**Background:**

In industrialized countries, improvements have been made in both maternal and newborn health. While attention to antenatal care is increasing, excessive medicalization is also becoming more common.

The aim of this study is to compare caesarean section (CS) frequency and ultrasound scan utilization in a public model of care involving both midwives and obstetricians with a private model in which care is provided by obstetricians only.

**Methods:**

Design: Observational population-based study. Setting: Reggio Emilia Province. Population: 5957 women resident in the province who delivered between October 2010 and November 2011. Main outcome measures: CS frequency and ultrasound scan utilization, stillbirths, and other negative perinatal outcomes. Women in the study were searched in the public family and reproductive health clinic medical records to identify those cared for in the public system. Outcomes of the two antenatal care models were compared through multivariate logistic regression adjusting for maternal characteristics and, for CS only, by stratifying by Robson’s Group.

**Results:**

Compared to women cared for in private services (N = 3,043), those in public service (N = 2,369) were younger, less educated, more frequently non-Italian, and multiparous. The probability of CS was slightly higher for women cared for by private obstetricians than for those cared for in the public system (31.8% vs. 27.1%; adjusted odds ratio: 1.10; 95% CI: 0.93–1.29): The probability of having more than 3 ultrasound scans was higher in private care (89.6% vs. 49.8%; adjusted odds ratio: 5.11; 95% CI: 4.30–6.08). CS frequency was higher in private care for all Robson’s classes except women who underwent CS during spontaneous labour. Among negative perinatal outcomes only a higher risk of pre-term birth was observed for pregnancies cared for in private services.

**Conclusions:**

The public model provides less medicalized and more guidelines-oriented care than does the private model, with no increase in negative perinatal outcomes.

## Background

Although pregnancy is a physiological process, the mother’s and/or newborn’s health can at times be at risk. In industrialized countries, improvements have been made in both maternal and newborn health. While attention to antenatal care is increasing, excessive medicalization is also becoming more common.

The rate of caesarean sections (CS) is increasing in all industrialized countries [1]. Despite the recommendations of the World Health Organization (WHO) [2,3] and of the Italian government [4] to reduce CS, the percentage of CS in Italy has risen from 11% in 1980 [5] to 37.5% in 2010 [6].

The rising CS rate can be attributed to a range of factors that include the perception that CS is a safe procedure, the lack of awareness of its possible adverse consequences, an increasing proportion of complicated pregnancies, medicolegal concerns, and women’s preferences [7].

The use of diagnostic procedures is increasing as well. Italian national guidelines indicate that only two ultrasound scans (US) should be performed [8], with only three US scans provided free of charge by the health system [9]. Nevertheless, in 2010 we observed an average of 5.3 ultrasound scans per birth, for physiological and pathological pregnancies alike [6].

Midwives, obstetricians, and general practitioners are recognized around the world as having the expertise to offer antenatal care. Efficacy and effectiveness evaluations of different models of care have been conducted: for example, two Cochrane Systematic Reviews of RCTs report several benefits to mothers and babies of midwife-led care and have not identified any adverse effects compared to other models of care [10,11].

Although the vast majority of women in Italy are cared for by private obstetricians [12,13], health authorities are nevertheless promoting public care in family and reproductive health clinics with a new model of care for physiological pregnancies based on midwives [14].

In 2006, the provincial family and reproductive health clinics of Reggio Emilia established that responsibility in pathological and physiological situations would be shared between midwives and obstetricians, with the former playing a central role in physiological pregnancy care. Private antenatal care in Reggio Emilia, instead, is administered almost exclusively by an obstetrician, as is the case in the rest of Italy as well [12,13].

This study takes into consideration two models of antenatal care: the public health model involving both midwives and obstetricians and the private model, in which care is offered by obstetricians only.

The objective of this study was to compare the two models of care in terms of the medicalization of pregnancy, in particular caesarean section frequency and ultrasound test utilization. We also compared the occurrence of negative perinatal outcomes in the two models of care as possible side effects of the reduction of pregnancy medicalization.

## Methods

### Study design

This is a population-based observational study based on routinely collected data and clinical record linkage.

### Study population

The study considered all deliveries between 01 October 2010 and 30 November 2011 of women resident in the Province of Reggio Emilia, Emilia-Romagna Region, who were registered in the Birth Certificate (BC) database. All pregnancies are included except for those of women resident in the one district (Castelnuovo ne Monti) where model of care involving both midwives and obstetricians was not available during the study period.

### Setting and description of intervention

In 2011, the Province of Reggio Emilia, Emilia-Romagna, Italy, had a resident population of around 530,300, with nearly 5,500 births. There are 6 health districts and a total of 21 family and reproductive health clinics in the province; at the time of the study, one district (Castelnuovo ne Monti), with one family and reproductive health clinic, had not yet implemented midwife pregnancy care. As previously reported, the 288 births from resident women in this district during the study period were therefore excluded from the analyses.

Women in the province can choose to be cared for either by these public family and reproductive health clinics for free or by obstetricians in private practice, for a fee.

In the family and reproductive health clinics women are seen by a midwife who, together with an obstetrician, determines whether or not the pregnancy is physiological. When it is, the midwife is responsible for the woman, scheduling and conducting check ups, monitoring laboratory tests, scheduling ultrasound tests (which are performed by the obstetrician), and keeping the woman updated throughout her pregnancy. If the pregnancy is not physiological, the midwife and the obstetrician share the caseload. In the province of Reggio Emilia, antenatal care usually is transferred between the 38^th^ and the 40^th^ week of gestation, depending on the health district, from the family and reproductive health clinic to the hospital antenatal clinic.

In the private model of care, the obstetrician alone is responsible for care and follows his/her own procedures. At term, women in private care are also referred to the hospital antenatal clinic, but their obstetrician can care for them during this phase as well.

### Data sources

Three different databases were used in this study.

Birth Certificate (BC) database: it includes all the deliveries taking place both in hospitals and in other health facilities. Information is collected, through an analysis of the medical record and an interview of the mother conducted by the midwife, on antenatal care, maternal characteristics, newborn characteristics, and delivery procedures. Reporting is compulsory for all births in hospitals (private and public) [15].

Family and reproductive health clinical records archive: information is collected on the procedures performed during antenatal care in the public clinics. Although clinical record registration was already in place before this study, its registration modalities were redefined and standardised throughout the districts at the beginning of the study period.

Hospital Discharge database: information is collected on all the hospital admissions and discharges occurring in all Italian public and private hospitals.

### Exposure assessment: definition of the model of antenatal care

All the pregnancies included in the study were linked to the family and reproductive health clinic records opened in 2010 and in 2011 by using the mother’s name, date of birth, and unique identification number. Those successfully linked were considered as cared for in the public family and reproductive health clinics; those who did not link were considered as cared for by obstetricians in private practice. The information on antenatal care reported in the BC database was also considered: if record linkage results were not consistent with the information on the model of care reported in the BC database, women were considered as not classifiable and were analyzed separately.

### Outcomes definition

Caesarean section frequency was considered a dichotomous variable (CS vs all other vaginal deliveries), as was the number of ultrasound scans during pregnancy (having 3 or more than 3 ultrasound scans); number of ultrasound scans was self-reported. Concerning the occurrence of negative perinatal outcomes, birth weight, birth outcome (live/stillbirth), Apgar Score, and need of intensive care were analyzed. All outcomes were retrieved from the BC.

### Maternal characteristics

All of the following maternal characteristics were collected from the BC database: educational level (high - university level: ≥16 years of education; medium - secondary level: 13-16 years of education, low level: <13 years of education), maternal age (five-year age groups), nationality (non-Italians vs Italians, based on citizenship), and parity (nulliparous vs multiparous women).

### Pregnancy, delivery, and newborn characteristics

Information on pregnancy, delivery, and newborn characteristics were collected from the BC database. Regarding pregnancy and delivery, the course of pregnancy (physiological or pathological, according to a comprehensive evaluation made by the midwife after completing the birth certificate), the type of reproduction (assisted or not assisted), at least one previous caesarean section, the type of pregnancy (single or multiple), the gestational age, the foetal presentation, the hospital in which the woman delivered, the number of check ups during pregnancy, the gestational age at the first check up, and attendance of antenatal classes were considered.

In order to examine more clinically relevant groupings for CS rates and compare public and private antenatal care, an analysis based on Robson Ten Group Classification Scheme (RTGCS) [16,17] was undertaken. Robson classifies women in ten groups according to parity, past obstetric history, singleton or multiple pregnancy, foetal presentation, gestational age, and mode of onset of labour/delivery. This classification has been shown to be useful for monitoring CS rates and their components, allowing risk stratification [18-22]. Of CS before labour, elective CS were identified through a dedicated variable in the BC database.

Hospitalization during pregnancy was also ascertained through record linkage with the Hospital Discharge database (using a personal identification number); this information was used to further rule out non-physiological pregnancies.

### Statistical analyses

Sociodemographic, pregnancy, and delivery characteristics were compared between the two models of care, with differences between public and private care tested with chi2 test. Furthermore, differences between Italian and non-Italian women were analysed.

Outcomes were compared between the antenatal care models through multivariate logistic regression models adjusting for maternal characteristics. The following covariates were chosen *a priori:* educational level, maternal age, citizenship, parity, course of pregnancy (pathological or physiological), gestational age (as continuous variable in weeks, after checking for linearity of the link with CS), hospitalization during pregnancy (none/at least one), and hospital of delivering (only to model the risk of CS). The same analyses were conducted excluding both non-Italians, because they were cared for almost exclusively in public care, and non-physiological pregnancies, i.e. those classified as pathological in the BC or that required hospitalization before delivery, because only physiological pregnancies can be cared for exclusively by the midwife in the public model of care.

Proportions of CS by Robson Groups and the relative size of each group were presented and compared with regard to the two models of care. To compare the risk of CS in the two models of care by each Robson class, we present odds ratios adjusted for maternal age, educational level, and citizenship.

We compared low birth weight rate (<=2500 g), stillbirth rate, pre-term birth rate (<37 weeks), need of intensive care, and Apgar score of the two groups through separate logistic regression models adjusting for citizenship and gestational age. For birth weight, we restricted the analysis only to term births and Italians.

Data analysis was performed using Stata 11.0.

### Details of ethics approval

Definition and piloting of pregnancy indicators was required by Regional Health Authority in 2008 [Giunta della Regione Emilia-Romagna “Direttiva alle aziende sanitarie in merito al programma percorso nascita” Delibera di giunta n.ro 2008/533]. The individual data contained in the database were analyzed only after anonymization. The Local Health Authority Medical Administration commissioned the present research and authorized its publication, exempting it from Ethical Review Board approval.

## Results

**Figure 1:**
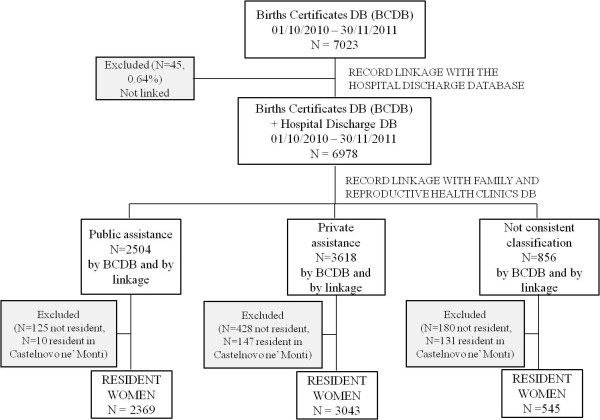
Number of women in the study and assistance characteristics.

After the record linkage between the Birth Certificate (BC) database and the Hospital Discharge database, 6978 childbirths were identified. Through the comparison between the categorization of model of care deduced by record linkage and the one registered in the BC database, three different exposure groups were defined (Figure 1).

Sociodemographic differences between women in the different models of care were observed (Table 1). Women cared for in public family and reproductive health clinics, compared with those cared for by a private obstetrician, were younger, less educated, more frequently non-Italian, and multiparous. There were also differences in pathological pregnancies (11.3% vs 9.0% in public and private care, respectively), frequency of hospitalization during pregnancy (12.0% vs 10.1%), assisted reproduction (0.5% vs 3.2%), previous caesarean section (22.2% vs 23.6%), and multiple pregnancies (1.1% vs 2.2%).

**Table 1:** Sociodemographic characteristics of women and characteristics of pregnancies and deliveries by models of care

	**Model of care**							
	**Public**		**Private**			**Unclassifiable**		**Total**
	**N**	**%**	**N**	**%**	**chi**^**2 **^p-value*	**N**	**%**	**N**
*Age (by class)*								
15-19	54	2.3	16	0.5		17	3.1	87
20-24	419	17.7	132	4.3		65	11.9	616
25-29	751	31.7	613	20.1		133	24.4	1,497
30-34	672	28.4		1,127	37.0		182	33.4	1,981
35-39	384	16.2		911	29.9		125	22.9	1,420
40+	89	3.8		244	8.0	p < 0.00005	23	4.2	356
*Educational level*									
High	309	13.0		949	31.2		96	17.6	1,354
Medium	836	35.3		1,558	51.2		243	44.6	2,637
Low	1,224	51.7		536	17.6	p < 0.00005	206	37.8	1,966
*Nationality*									
Italian	866	36.6		2,857	93.9		356	65.3	4,079
Non-Italian	1,503	63.4		186	6.1	p < 0.00005	189	34.7	1,878
*Parity*									
Nulliparous	975	41.2		1,659	54.5		286	52.5	2,920
Multiparous	1,394	58.8		1,384	45.5	p < 0.00005	259	47.5	3,037
*Course of pregnancy*									
Physiological	2,101	88.7		2,767	90.9		469	86.1	5,337
Pathological	268	11.3		275	9.0	#p = 0.006	76	13.9	619
Missing	0	0.0		1	0.0		0	0.0	1
*Hospitalization during pregnancy*									
None	2,085	88.0		2,736	89.9		473	86.8	5,294
At least one	284	12.0		307	10.1	p = 0.026	72	13.2	663
*Assisted reproduction*									
Yes	12	0.5		98	3.2		11	2.0	121
No	2,356	99.5		2,945	96.8	#p < 0.00005	534	98.0	5,835
Missing	1	0.0		0	0.0		0	0.0	1
*Previous caesarean section*^**§**^									
None	1,084	77.8		1,057	76.4		181	69.9	2,322
At least one	310	22.2		327	23.6	p = 0.38	78	30.1	715
*Type of pregnancy*									
Single	2,342	98.9		2,976	97.8		530	97.2	5,848
Multiple	25	1.1		67	2.2		14	2.6	106
Missing	2	0.1		0	0.0	p = 0.0012	1	0.2	3
*Gestational age (weeks)*									
<37	152	6.4		255	8.4		62	11.4	469
> = 37	2,214	93.5		2,781	91.4	#p = 0.006	480	88.1	5,475
Missing	3	0.1		7	0.2		3	0.6	13
*Newborn presentation*									
Cephalic	2,251	94.1		2,863	94.1		508	93.2	5,622
Other presentations	118	5.9		180	5.9	p = 0.14	37	6.8	62
*Number of check ups*									
<=3	110	4.6		31	1.0		32	5.9	173
>3	2,259	95.4		3,012	99.0	#p < 0.00005	512	93.9	5,783
Missing	0	0.0		0	0.0		1	0.2	1
*Gestational age at first check up (week)*									
<=12	1,853	78.2		2,858	93.9		460	84.4	5,171
>12	512	21.6		179	5.9	#p < 0.00005	83	15.2	774
missing	4	0.2		6	0.2		2	0.4	12
*Antenatal classes*									
No	1,967	83.0		1,928	63.4		405	74.3	4,300
Yes, public family clinics	335	14.1		519	17.1		77	14.1	931
Yes, public hospital	32	1.4		493	16.2		49	9.0	574
Yes, private structure	2	0.1		93	3.1	#p < 0.00005	6	1.1	101
Missing	33	1.4		10	0.3		8	1.5	51
*Hospital*									
LHU Montecchio	322	13.6		293	9.6		39	7.2	654
LHU Guastalla	401	16.9		393	12.9		70	12.8	864
LHU Scandiano	430	18.2		217	7.1		72	13.2	719
LHU Castelnovo ne’ Monti	2	0.1		10	0.3		5	0.9	17
Hospital Santa Maria Nuova	967	40.8		1,361	44.7		217	39.8	2,545
Hospitals outside the province	247	10.4		769	25.3	p < 0.00005	142	26.1	1,158
***Outcome***									
*Mode of delivery*									
Vaginal not operative	1,620	68.4		1,938	63.7		331	60.7	3,889
Vaginal with forceps	2	0.1		0	0.0		0	0.0	2
Vaginal with suction cup	105	4.4		138	4.5		22	4.0	265
Caesarean section	642	27.1		967	31.8	p = 0.0002	192	35.2	1,801
*Number of ultrasound scans*									
0-2	110	4.6		17	0.6		27	5.0	154
3	1,080	45.6		298	9.8		140	25.7	1,518
> = 4	1,179	49.8		2,726	89.6	#p < 0.00005	377	69.2	4,282
Missing	0	0.0		2	0.1		1	0.2	3
Total	2,369	100.0		3,043	100.0		545	100.0	5,957

^**§**^only multiparous women were considered.

*probability of equal distribution in public and private care, chi2.

#Missing values are excluded.

The percentage of women with fewer than 3 check ups during pregnancy, late presentation at first check up (after the 12^th^ week of gestation), and who did not attend antenatal classes was higher in the public service. Non-Italian women more frequently had fewer than three check ups, (76.6% vs 23.4% non-Italians and Italians, respectively), less frequently attended antenatal classes (71.5% vs. 28.5%), and presented after 12^th^ week (85.6% vs. 14.4%). The higher proportion of non-Italian women cared for by the public health service accounted for all the differences in these variables between the two models of care.

Caesarean sections were less frequent in women assisted by the public family and reproductive health clinics than in those assisted by a private obstetrician (27.1% vs 31.8%). In the public health service, 45.6% of the women had 3 ultrasound scans; in private practice, this percentage was 9.8%. The percentage of non-Italian women having fewer than 3 ultrasound scans in the public health service was nearly 87%.

The proportion of low birth weight did not differ between the two groups (Table 2, chi2 p = 0.82; odds ratio for being < =2500 g restricted to Italians and full term pregnancy and adjusted by gestational age private vs. public 0.76, 95% CI 0.49-1.16); the same was found for Apgar (Table 2, chi2 p = 0.27; odds ratio of having 6 or less Apgar score, adjusted for gestational age and citizenship private vs. public 0.74 95% CI 0.30-1.85) and intensive care (Table 2 chi2 p = 0.97; odds ratio of receiving intensive care, adjusted for gestational age and citizenship private vs. public 1.00 95% CI 0.63-1.59). Stillbirths were only slightly higher in public health service though the difference was not significant (Table 2 chi2 p = 0.3; odds ratio adjusted for citizenship and gestational age private vs. public 0.41; 95% CI 0.11-1.56). In both groups, 60% of stillbirths occurred in cases of pathological pregnancies. In the public health services, 5 stillbirths out of 8 were to a non-Italian mother; in private care, all stillbirths had an Italian mother. The rate is almost identical in the two settings when we look only at Italians: 3/880 in public health care vs 6/3070 in private care (adjusted odds ratio 0.40; 95% CI 0.09-1.71). A significant difference was observed only in terms of the length of pregnancy: preterm babies were more frequent in women cared for by private obstetricians (chi2 p = 0.006, adjusted odds ratio 1.34; 95% CI 1.08-1.65) (Table 1).

**Table 2:** Newborn characteristics by models of care

	**Model of care**							
	**Public**		**Private**			**Unclassifiable**		**Total**
	**N**	**%**	**N**	**%**	chi2 p-value*	**N**	**%**	**N**
*Birth weight (gr)*								
> = 2500	2,224	92.9	2,872	92.3		502	89.8	5,598
1500-2499	145	6.1	207	6.7		47	8.4	399
1000-1499	15	0.6	18	0.6		8	1.4	41
<1000	11	0.5	13	0.4	p = 0.82	2	0.4	26
*Vitality*								
Live birth	2,387	99.7	3,104	99.8		554	99.1	6,045
Stillbirth	8	0.3	6	0.2	p = 0.3	5	0.9	19
Apgar score*								
7-10	2,370	99.3	3,089	99.5		549	99.1	6,008
4-6	16	0.7	14	0.5	§p = 0.27	5	0.9	35
1-3	1	0.0	1	0.0		0	0.0	2
Intensive care*								
Yes, manual ventilation	35	1.5	54	1.7		8	1.4	97
Yes, intubation	20	0.8	18	0.6		6	1.1	44
No	2,332	97.7	3,032	97.7	#p = 0.97	540	97.5	5,904
Total	2,395	100.0	3,110	100.0		559	100.0	6,064

*Stillbirths are excluded.

§Apgar 7-10 vs. 1-6.

#No vs. any intervention.

Table 3 presents the results of the logistic regression models. Women in the private model showed a slightly higher probability of CS than did women in the public health service. Excluding non-physiological pregnancies, i.e. those classified as pathological in the BC or that required hospitalization, and Italian women, the risk for CS in private care was higher and borderline significant (OR: 1.16 95% CI: 0.93 – 1.44). Results of the analysis of the number of ultrasound scans indicated a higher risk of receiving more than three ultrasound scans for women in the private model of care.

**Table 3:** Adjusted OR of CS and adjust OR of more than 3 ultrasound scans and 95% CI

**A**	**Caesarean section (N = 5,957)**				**Ultrasound scan (N = 5,787)**			
	**N women**	**N**	OR*	**95% CI**	**N women**	**N**	OR**	**95% CI**
**Model of care**								
Public	2,369	642	1 (Ref.)		2,259	1,179	1 (Ref.)	
Private	3,043	967	1.10	(0.93 - 1.29)	3,024	2,726	5.11	(4.30 – 6.08)
Not classifiable	545	192	1.30	(1.04 - 1.61)	517	377	1.94	(1.55 - 2.42)
**B**	**Caesarean section (N = 3,383)**				**Ultrasound scan (N = 3,144)**^**§**^			
	**N women**	**N**	OR*	**95% CI**	**N women**	**N**	OR**	**95% CI**
**Model of care**								
Public	695	169	1 (Ref.)		632	382	1 (Ref.)	
Private	2403	697	1.16	(0.93 - 1.44)	2260	2056	5.92	(4.73 - 7.41)
Not classifiable	285	86	1.28	(0.93 - 1.78)	259	207	2.52	(1.77 - 3.58)

*Adjusted for maternal characteristics: educational level, maternal age, parity, hospital, gestational age and, only in model A, course of pregnancy, hospitalization during pregnancy and citizenship.

**Adjusted for maternal characteristics: educational level, maternal age, parity, gestational age, and, only in model A, course of pregnancy, hospitalization during pregnancy and citizenship.

^**§**^Only women with the first check up before the 12° week of gestation were considered.

A: all women included; B: only physiological pregnancies, Italian women and women without hospitalizations during pregnancy.

Table 4 shows the rates of CS births by Robson’s 10-group classification and the adjusted OR of CS for each class. Most births, but not most CS, occurred in groups 1 and 3, which include single cephalic, full-term pregnancies with spontaneous labour women (Group 1) and multiparous women with no uterine scar (Group 3). CSs in these groups are generally performed as emergencies. In both groups, the adjusted OR for maternal age, educational level, and citizenship indicated a lower risk of CS in private compared to public care, though the differences were not statistically significant. Groups 2a and 4a include single cephalic, full-term pregnancies with induced labour in nulliparous women (Group 2a) and in multiparous women without uterine scar (Group 4a). Groups 2b and 4b include single cephalic, full-term pregnancies with a CS before labour onset in nulliparous women (Group 2b) and in multiparous women without uterine scar (Group 4b). Considering nulliparous and multiparous together, the percentage of women with a caesarean section before labour was 3.4% in public health service and 7.0% in private care. Taking into account only elective CS, these percentages were 1.7% and 5.4%, respectively. The results were substantially the same when the analyses are restricted to only physiological pregnancies.

**Table 4:** Rates of CS by Robson classification and model of care, and adjusted OR of CS

		**Public**				**Private**				**Private vs public**
**Robson groups**	**Description**	**N women**	**Proportion of all women (%)**	**N Caesarean Sections (CS)**	**CS rate (x100)**	**N women**	**Proportion of all women (%)**	**N Caesarean Sections (CS)**	**CS rate (x100)**	**Adjusted OR**^**§ **^** (95% CI)**
1	Nulliparous, single cephalic, > = 37 weeks, in spontaneous labour	462	19.5	40	8.7	831	27.4	68	8.2	0.60 (0.35 - 1.02)
2a	Nulliparous, single cephalic, > = 37 weeks, induced labour	331	14.0	81	24.5	428	14.1	115	26.9	1.64 (1.14 - 2.34)
2b	Nulliparous, single cephalic, > = 37 weeks, CS before labour	53	2.2	53	100.0	141	4.6	141	100.0	
3	Multiparous (excluding prev. CS), single cephalic, > = 37 weeks, in spontaneous labor	704	29.8	15	2.1	695	22.9	9	1.3	0.42 (0.13 - 1.30)
4a	Multiparous (excluding prev. CS), single cephalic, > = 37 weeks, induced labour	256	10.8	18	7.0	186	6.1	10	5.4	1.66 (0.94 – 2.91)
4b	Multiparous (excluding prev. CS), single cephalic, > = 37 weeks, CS before labour	29	1.2	29	100.0	73	2.4	73	100.0	
5	Previous CS, single cephalic, > = 37 weeks	268	11.3	232	86.6	278	9.2	253	91.0	1.56 (0.78 - 3.13)
6	All nulliparous breeches	47	2.0	45	95.7	86	2.8	85	98.8	-
7	All nulliparous breeches (including prev. CS)	37	1.6	37	100.0	42	1.4	42	100.0	-
8	All multiple pregnancies (including prev. CS)	25	1.1	21	84.0	67	2.2	60	89.6	-
9	All abnormal lies (including prev. CS)	28	1.2	9	32.1	25	0.8	13	52.0	-
10	All single cephalic, <=36 weeks (including prev. CS)	124	5.2	61	49.2	185	6.1	96	51.9	0.93 (0.51 - 1.71)
Total		2,364	641	100.0	27.1	3,037	965	100.0	31.8	

Eleven births could not be classified according to RTGCS because of missing data.

^§^Adjusted for maternal characteristics: educational level, maternal age, citizenship, hospital.

Considering classes 2 and 4 on the whole, the adjusted OR indicated a higher risk of CS for women in private care (OR: 1.64 95% CI: 1.14-2.34; OR: 1.66 95% CI: 0.94-2.91 for classes 2 and 4, respectively; restricting the analysis only to class 2a and 4a the ORs are 1.43 95% CI 0.93-2.20 and 0.53 95% CI 0.18-1.58, respectively).

Group 5 consisted of single cephalic, full-term pregnancies in women with one or more uterine scars. This group accounted for nearly 10% of births. CS rates were high in this group and contributed most to the overall CS rates in both models. The proportion of CS was higher in private care than in public care although not significantly so after adjustment (OR: 1.56 95% CI: 0.78-3.13). Groups 6-9 included breech presentations in nulliparous (Group 6) and multiparous (Group 7) women, all multiple pregnancies (Group 8), and abnormal lies (Group 9). These groups accounted for the minority of births. CS rates were very high, particularly in private care. Due to the small numbers, multivariate models were not used. The CS rate in Group 10 (preterm births) was nearly 50% for both the public and private models of care.

Among the women cared for in the family and reproductive health clinics, it was possible to distinguish between women who were cared for only by a midwife and those who were cared for by both a midwife and an obstetrician. In pregnancies exclusively supervised by the midwife, CSs were 17% and the percentage of women with only 3 ultrasound scans was more than 50% (Table 5). This analysis has been restricted to physiological pregnancies, since a woman can be assigned to midwifery care only if the pregnancy is physiological and has no complications or known risk factors.

**Table 5:** Number and percentage of CS and of women with 3 ultrasound scans for physiological pregnancy and for different models of care

**Physiological pregnancies**		**N**	**Caesarean section**		**Ultrasound scans**	
			**CS (N)**	**%**	**3 ultrasound scans (N)**	**%**
Public care	Midwife only	1051	184	17.5	591	56.2
	Midwife and obstetrician	1050	350	33.3	412	39.2
Private care		2767	817	29.5	284	10.3

## Discussion

### Main findings

The level of pregnancy medicalization in the study population is high compared to international standards, as is true for Italy in general [6,12,13]. We observed a slightly lower rate of caesarean sections and a dramatically lower ultrasound scan use in physiological pregnancies in public care provided collaboratively by midwives and obstetricians than in private care with obstetrician only. The public model provides less medicalized care without there being an increase in any of the negative perinatal outcomes studied.

### Strengths and limitations

Our population-based cohort study compares the outcomes in clinical practice of a public model adopting midwife-led antenatal care for physiological pregnancies and obstetrician-led care for pathological pregnancies with those of private, obstetrician-only care.

We know that the two groups are very different in many regards and that a strong self-selection bias acts in the choice between public, universally affordable care and more expensive private care. We therefore adjusted for all the known possible confounders. Unfortunately, other unknown socioeconomic, cultural, and behavioural factors, as well as not-collected clinical characteristics of women may be relevant to the studied outcomes and we cannot rule out that higher frequencies of women with such risk factors are cared for in one of the two models of care. Socioeconomic and cultural confounders probably contribute to reduce the risk of CS and increase the risk of adverse peri-natal outcome in women cared for in the public system. The direction of the confounding effect due to unknown clinical conditions cannot to be predicted, since some obstetric conditions identifiable during pregnancy and favouring CS, i.e. twins or assisted reproduction, are more frequent in private care than in the public, while other conditions, such as pathologic pregnancies, are more frequent in the public services. It is important to note that all the adjusting and outcome variables, including those regarding pathologies during pregnancy, were collected using an information source independent from the antenatal care setting, i.e. the BC database, based on postpartum hospital interviews, and hospital admissions.

In this study, it was not easy to interpret the role of midwives in reducing medicalization. In fact, we must consider that women cared for only by midwives are, by definition, the healthiest women, with no pregnancy risk factor. Therefore, the results reported in Table 5 are a result of selection bias in the group assigned to midwifery care that cannot be adjusted for. The only way to avoid this bias was to compare the whole group of women cared for in the public family and reproductive health clinics with the whole group of women cared for by private obstetricians.

In the study, it was impossible to assess the model of prenatal care for a group of women with discordant information in the two data sources. This group includes women who might have used both models of care, i.e., they were initially in the public service but then changed to the private model of care. This group may also include women referred to specialized care because of some pregnancy condition. The latter phenomenon may justify the higher risk of CS and the slightly worse neonatal outcomes.

### Interpretations

Users of the two models are very different: women who turn to the public family and reproductive health clinics are younger, more frequently non-Italian, have a low or medium educational level, and are quite frequently multiparous, as already observed in different Italian contexts [23-25]. Indeed, 60% of the women assisted in the public service are non-Italian. Foreign women experience more problematic antenatal care: their compliance to antenatal care is hampered by their socioeconomic conditions and lifestyles and they are characterized by poorer neonatal outcomes [13,23-27]. In Emilia-Romagna, the number of stillbirths [26,27] has been found to be higher in non-Italian women. Our data also found a relationship between the lower number of check ups, the lower attendance of antenatal classes, the higher frequency of presentation for the first check up after the 12^th^ week of gestation, and the higher percentage of women having fewer than 3 ultrasound scans and being non-Italian, as already observed in a nationwide sample survey [13]. Given these characteristics of immigrant women, analyses considering only Italians and physiological pregnancies were conducted; the results showed a stronger association between CS and private care and remained nearly unchanged when considering ultrasound scan.

The two Cochrane Systematic Reviews [10,11] on the effectiveness of different models of care underline that women cared for in midwife-led models of care were less likely to experience antenatal hospitalization, regional analgesia and intrapartum analgesia/anaesthesia, episiotomy, and instrumental delivery. Furthermore, they were less likely to experience foetal loss before 24 weeks’ gestation and their babies were more likely to have a shorter length of hospital stay. Even if there were no statistically significant differences in the overall estimates for caesarean births [10], two studies included in the Cochrane review, one conducted in Canada [28] and one in Australia [29], observed a lower CS rate for the midwife or midwife and obstetrician model of care compared to the standard physician-led model.

A population-based Italian survey also found that the risk of undergoing more than 3 US scans and CS was higher for women cared for by private gynaecologists compared to those cared for by public family and reproductive health clinics or midwives [13].

The analysis conducted by means of the Robson Classification system indicates that public care decreases the frequency of CS where recommendation for a CS is not absolute but, according to guidelines [4], a spontaneous delivery can be attempted. There is a higher probability of CS in classes 1 and 3, where CSs are mostly performed as emergencies. On the other hand, a higher probability of elective CS was found in private care for all classes when this was an option; the role of patient preferences and obstetric attitudes are obviously stronger in these cases [30].

Finally, it must be noted that the major non-clinical determinant of caesarean section in Italy [31,32] and in our own setting was the delivery hospital (see Additional file 1). This was expected, since the final decision of whether or not to perform a CS is the responsibility of the obstetrics team in the delivery room.

The differences between the two models of care are remarkable when considering ultrasound scan use, even when excluding non-physiological pregnancies. It must be noted that for these pregnancies the new guidelines [8] recommend only 2 ultrasound tests and state that there is no evidence of the efficacy of a late ultrasound scan (after 24 weeks of gestation) if there are no particular indications for it [33]. Private gynaecologists in Italy are usually paid directly by the women after each check up. The number of check ups and the number of procedures performed per check up, therefore, may be determined by market and patient satisfaction dynamics rather than by clinical appropriateness.

The Emilia-Romagna Region is the only region that has officially adopted the new Italian National Pregnancy Guidelines [4,8]. As our results show, public care deviates less from these recommendations compared with private care, thereby limiting the phenomenon of medicalization. More appropriateness and less medicalization mean more efficiency and, consequently, cost saving, which is an important consideration given the present difficult financial situation and its repercussions on the National Health Service. Moreover, the utilization of public health services will probably increase given that it is free of charge and that socioeconomic conditions are worsening for an ever-growing segment of the population.

## Conclusion

In conclusion, CS frequency was higher in private care for all Robson’s classes except for women who underwent CS during spontaneous labour. No increase in negative perinatal outcomes was observed. We also found an excess of ultrasound scans in private care, in contrast with national and international guideline standards. Furthermore, our results suggest that the diffusion and implementation of guidelines-based public care in Italy could reduce inappropriate use of ultrasound scans and could contribute to control the number of caesarean sections without causing any harm to newborns.

## Competing interests

The authors have no conflicts of interests to disclose.

## Authors’ contributions

BL, PGR, CS, EA and MA planned the study and defined the methods. BD, CM and LA upgraded and coordinated data collection in the family and reproductive health clinics in Reggio Emilia. BL conducted the statistical analyses. PGR, BL, and CS drafted the paper. All authors read and approved the final manuscript.

## Pre-publication history

The pre-publication history for this paper can be accessed here:

http://www.biomedcentral.com/1471-2393/14/72/prepub

## Supplementary Material

Additional file 1: Table A1 Adjusted OR of CS and 95% CI (Model A Table 3). **Table A2.** Adjusted OR of CS and 95% CI. Only Italian women, physiological pregnancies and women without hospitalization during pregnancy (Model B Table 3). **Table A3.** Adjusted OR of more than 3 ultrasound scans and 95% CI (Model A Table 3). **Table A4.** Adjusted OR of more than 3 ultrasound scans and 95% CI. Only Italian women, with physiological pregnancies, without hospitalization during pregnancy and with first check up before 12th week of gestation (Model B Table 3). Click here for file
