# Supplementary material for: Public and private pregnancy care in Reggio Emilia Province: an observational study on appropriateness of care and delivery outcomes
Source: BMC Pregnancy Childbirth. 2014 Feb 17;14:72. doi: 10.1186/1471-2393-14-72 (PMC4015597; doi:10.1186/1471-2393-14-72)
Supplement: Additional file 1: Table A1 — Adjusted OR of CS and 95% CI (Model A Table 3). Table A2. Adjusted OR of CS and 95% CI. Only Italian women, physiological pregnancies and women without hospitalization during pregnancy (Model B Table 3). Table A3. Adjusted OR of more than 3 ultrasound scans and 95% CI (Model A Table 3). Table A4. Adjusted OR of more than 3 ultrasound scans and 95% CI. Only Italian women, with physiological pregnancies, without hospitalization during pregnancy and with first check up before 12th week of gestation (Model B Table 3). [file 1471-2393-14-72-S1.doc]

TableA 1 Adjusted OR of CS and 95% CI (Model A Table 3)

|  | Odds Ratio | 95% CI | |
| --- | --- | --- | --- |
| *Model of care* |  |  |  |
| Public | 1(Ref.) |  |  |
| Private | 1.10 | 0.93 | 1.29 |
| Not classificable | 1.30 | 1.04 | 1.61 |
| *Educationale level* |  |  |  |
| High | 1(Ref.) |  |  |
| Medium | 1.20 | 1.03 | 1.40 |
| Low | 1.27 | 1.07 | 1.52 |
| *Maternal age (years)* |  |  |  |
| 15-19 | 0.46 | 0.23 | 0.92 |
| 20-24 | 1(Ref.) |  |  |
| 25-29 | 1.20 | 0.95 | 1.52 |
| 30-34 | 1.48 | 1.18 | 1.88 |
| 35-39 | 2.03 | 1.59 | 2.59 |
| 40+ | 2.66 | 1.96 | 3.63 |
| *Nationality* |  |  |  |
| Italians | 1(Ref.) |  |  |
| Foreigners | 1.11 | 0.95 | 1.31 |
| *Parity* |  |  |  |
| Nulliparous | 1(Ref.) |  |  |
| Multiparous | 0.91 | 0.80 | 1.03 |
| *Hospital* |  |  |  |
| LHU Montecchio | 1(Ref.) |  |  |
| LHU Guastalla | 0.90 | 0.71 | 1.13 |
| LHU Scandiano | 0.74 | 0.57 | 0.95 |
| LHU Castelnovo ne' Monti | 2.40 | 0.89 | 6.47 |
| Hospital Santa Maria Nuova | 0.94 | 0.77 | 1.15 |
| Hospitals outside the province | 1.17 | 0.94 | 1.46 |
| *Course of pregnancy* |  |  |  |
| Physiological | 1(Ref.) |  |  |
| Pathological | 1.26 | 1.04 | 1.54 |
| *Hospitalization during pregnancy* |  |  |  |
| none | 1(Ref.) |  |  |
| at least one | 1.20 | 1.00 | 1.45 |
| *Gestational age (weeks)* | 0.75 | 0.72 | 0.77 |

Table A 2 Adjusted OR of CS and 95% CI. Only Italian women, physiological pregnancies and women without hospitalization during pregnancy (Model B Table 3)

|  | Odds Ratio | 95% CI | |
| --- | --- | --- | --- |
| *Model of care* |  |  |  |
| Public | 1(Ref.) |  |  |
| Private | 1.16 | 0.93 | 1.44 |
| Not classificable | 1.28 | 0.93 | 1.78 |
| *Educationale level* |  |  |  |
| High | 1(Ref.) |  |  |
| Medium | 1.12 | 0.93 | 1.36 |
| Low | 1.41 | 1.11 | 1.78 |
| *Maternal age (years)* |  |  |  |
| 15-19 | 0.25 | 0.06 | 1.09 |
| 20-24 | 1(Ref.) |  |  |
| 25-29 | 1.08 | 0.72 | 1.62 |
| 30-34 | 1.45 | 0.98 | 2.15 |
| 35-39 | 1.95 | 1.31 | 2.91 |
| 40+ | 2.23 | 1.41 | 3.53 |
| *Parity* |  |  |  |
| Nulliparous | 1(Ref.) |  |  |
| Multiparous | 1.01 | 0.86 | 1.19 |
| *Hospital* |  |  |  |
| LHU Montecchio | 1(Ref.) |  |  |
| LHU Guastalla | 0.73 | 0.53 | 1.01 |
| LHU Scandiano | 0.66 | 0.47 | 0.92 |
| LHU Castelnovo ne' Monti | 1.74 | 0.51 | 5.96 |
| Hospital Santa Maria Nuova | 0.78 | 0.60 | 1.02 |
| Hospitals outside the province | 1.02 | 0.77 | 1.35 |
| *Gestational age (weeks)* | 0.72 | 0.68 | 0.76 |

Table A 3 Adjusted OR of more than 3 ultrasound scans and 95% CI (Model A Table 3)

|  | Odds Ratio | 95% CI | |
| --- | --- | --- | --- |
| *Model of care* |  |  |  |
| Public | 1(Ref.) |  |  |
| Private | 5.11 | 4.30 | 6.08 |
| Not classificable | 1.94 | 1.55 | 2.42 |
| *Educationale level* |  |  |  |
| High | 1(Ref.) |  |  |
| Medium | 0.99 | 0.82 | 1.20 |
| Low | 0.75 | 0.61 | 0.91 |
| *Maternal age (years)* |  |  |  |
| 15-19 | 1.05 | 0.63 | 1.74 |
| 20-24 | 1(Ref.) |  |  |
| 25-29 | 1.12 | 0.90 | 1.40 |
| 30-34 | 1.21 | 0.97 | 1.52 |
| 35-39 | 1.37 | 1.07 | 1.76 |
| 40+ | 1.84 | 1.26 | 2.69 |
| *Nationality* |  |  |  |
| Italians | 1(Ref.) |  |  |
| Foreigners | 0.52 | 0.45 | 0.61 |
| *Parity* |  |  |  |
| Nulliparous | 1(Ref.) |  |  |
| Multiparous | 0.85 | 0.74 | 0.98 |
| *Course of pregnancy* |  |  |  |
| Physiological | 1(Ref.) |  |  |
| Pathological | 2.24 | 1.73 | 2.89 |
| *Hospitalization during pregnancy* |  |  |  |
| none | 1(Ref.) |  |  |
| at least one | 1.19 | 0.95 | 1.48 |
| *Gestational age (weeks)* | 1.01 | 0.97 | 1.05 |

Table A 4 Adjusted OR of more than 3 ultrasound scans and 95% CI. Only Italian women, with physiological pregnancies, without hospitalization during pregnancy and with first check up before 12^th^ week of gestation (Model B Table 3)

|  | Odds Ratio | 95% CI | |
| --- | --- | --- | --- |
| *Model of care* |  |  |  |
| Public | 1(Ref.) |  |  |
| Private | 5.92 | 4.73 | 7.41 |
| Not classificable | 2.52 | 1.77 | 3.58 |
| *Educationale level* |  |  |  |
| High | 1(Ref.) |  |  |
| Medium | 1.00 | 0.77 | 1.30 |
| Low | 0.69 | 0.51 | 0.93 |
| *Maternal age (years)* |  |  |  |
| 15-19 | 1.14 | 0.47 | 2.77 |
| 20-24 | 1(Ref.) |  |  |
| 25-29 | 1.32 | 0.86 | 2.02 |
| 30-34 | 1.37 | 0.90 | 2.07 |
| 35-39 | 1.50 | 0.98 | 2.32 |
| 40+ | 2.77 | 1.49 | 5.15 |
| *Parity* |  |  |  |
| Nulliparous | 1(Ref.) |  |  |
| Multiparous | 0.85 | 0.68 | 1.05 |
| *Gestational age (weeks)* | 0.97 | 0.90 | 1.04 |
